# Outcomes improvement despite continuous visits of severely injured patients during the COVID-19 outbreak: experience at a regional trauma centre in South Korea

**DOI:** 10.1186/s12873-022-00726-1

**Published:** 2022-10-06

**Authors:** Sooyeon Kang, Ji Eun Park, Ji Wool Ko, Myoung Jun Kim, Young Un Choi, Hongjin Shim, Keum Seok Bae, Kwangmin Kim

**Affiliations:** 1 Department of Medicine, Yonsei University Wonju College of Medicine, Wonju, 26426 Korea; 2 Department of Surgery, Yonsei University Wonju College of Medicine, Ilsan-ro 20, 26426 Wonju, Gangwon-do Republic of Korea; 3 Regional Trauma Center, Wonju Severance Christian Hospital, Ilsan-ro 20, 26426 Wonju, Gangwon-do Republic of Korea; 4 Wonju Severance Pelvic Bone Research Group, Yonsei University Wonju College of Medicine, Ilsan-ro 20, 26426 Wonju, Gangwon-do Republic of Korea; 5 Center of Evidence Based Medicine, Institute of Convergence Science, Yonsei University, Seoul, 03722 Korea

**Keywords:** Outcomes improvement, COVID-19 outbreak, Severely injured patients

## Abstract

**Background:**

Understanding the changes in characteristics of patients who visited trauma centres during the coronavirus disease 2019 (COVID-19) pandemic is important to facilitate aneffective response. This retrospective study was conducted to analyse differences in the characteristics and outcomes of patients who visited our trauma centre between pre-COVID-19 and COVID-19 eras.

**Methods:**

Medical data of trauma patients enrolled in the Korean trauma database from 1 January 2018 to 31 August 2021 were collected. The number of trauma centre visits, patient characteristics, factors associated with in-hospital intervention, and outcomes werecompared between patients in the two time periods. Propensity score matching was performed to analyse the outcomes in patients with similar characteristics and severitybetween patients in the two time periods.

**Results:**

The number of emergency department (ED) trauma service visits reduced in the COVID-19 era. Based on the mean age, the patients were older in the COVID-19 era. Abbreviated injury scale (AIS) 1, AIS3, AIS5, and injury severity score (ISS) were higher in the COVID-19 era. The proportion of motor vehicle collisions decreased, whereas falls increased during the COVID-19 era. Ambulance transportation, admission to the general ward, and time from injury to ED visit significantly increased. Patient outcomes, such as hospital length of stay (LOS), intensive care unit (ICU) LOS, and duration of mechanical ventilation improved, while injury severity worsened during the COVID-19 era. After adjusting for patient characteristics and severity, similar findings were observed.

**Conclusion:**

The small reduction in the number of trauma patients and visits by patients who hadhigher ISS during the COVID-19 pandemic highlights the importance of maintaining trauma service capacity and capability during the pandemic. A nationwide or nationalmulticentre study will be more meaningful to examine the impact of the COVID-19 outbreak on the changes in trauma patterns, volume, and patient outcomes.

**Supplementary Information:**

The online version contains supplementary material available at 10.1186/s12873-022-00726-1.

## Introduction

Coronavirus disease 2019 (COVID-19) is an infectious disease caused by the severe acute respiratory syndrome coronavirus 2 (SARS-CoV-2) and was first reported in Wuhan, Hubei province, China, in December 2018 [1]. The virus, which can spread through direct contact, aerosols, air, and droplets, mainly affects the upper respiratory tractand can cause pneumonia in severe cases. This virus is highly contagious and has spread globally in a short time. Finally, on 11 March 2020, the World Health Organization declared COVID-19 a pandemic [2]. Due to the widespread COVID-19 cases, many countries declared a state of emergency and recommended partial or full restrictions on social activities.

The first case of COVID-19 in the Republic of Korea was reported on 20 January 2020. Korea experienced the COVID-19 outbreak earlier than that seen in many othercountries, starting on 18 February 2020. On 2 March 2020, the number of confirmedcases in Korea was 4,212, the second highest after China [3]. However, the curve flattened as the Korean government implemented policies to reduce the transmission ofthe disease. As of 23 July 2020, the number of confirmed cases was approximately 14,000. Since then, the number of confirmed COVID-19 cases has continued to increase; as of 31 August 2021 the cumulative number of confirmed cases was 253,445 [4]. To control the spread of COVID-19, social distancing measures were implemented,and the crisis alert level increased incrementally as the number of confirmed COVID-19 cases increased. Strict social distancing policies including restrictions on cafesandrestaurants after 21:00 and a ban on gatherings of more than four people, continued until August 2021.

The COVID-19 pandemic has undoubtedly disrupted essential health services and affected the incidence and mortality of other diseases. Trauma is one of the leading causes of death in the economically active population of Korea [5]. Despite social restriction policies by the government, patients still experience trauma and require significant health care resources for management. Understanding the changes in patients whovisited trauma centres during the COVID-19 pandemic is important to facilitate an effective response. Although many studies have addressed the impact of COVID-19 on trauma centre visits, they were conducted in the early stages of the COVID-19 pandemic and did not comprehensively analyse the clinical features of the patients, including patient outcomes [6–10].

This retrospective study was aimed to analyse the differences in the characteristics and outcomes of patients who visited our trauma centre between the pre-COVID-19 and COVID-19 eras. The primary hypothesis was that patient outcomes would be worseduring the outbreak than those before. A secondary hypothesis was that the number of trauma patients visiting the trauma centre would be lower during the outbreak thanthat previously. The last hypothesis was that the COVID-19 outbreak would result in changes of the characteristics of the patients who visited the trauma center.

## Materials and methods

### Patient selection and data collection

**Fig. 1:**
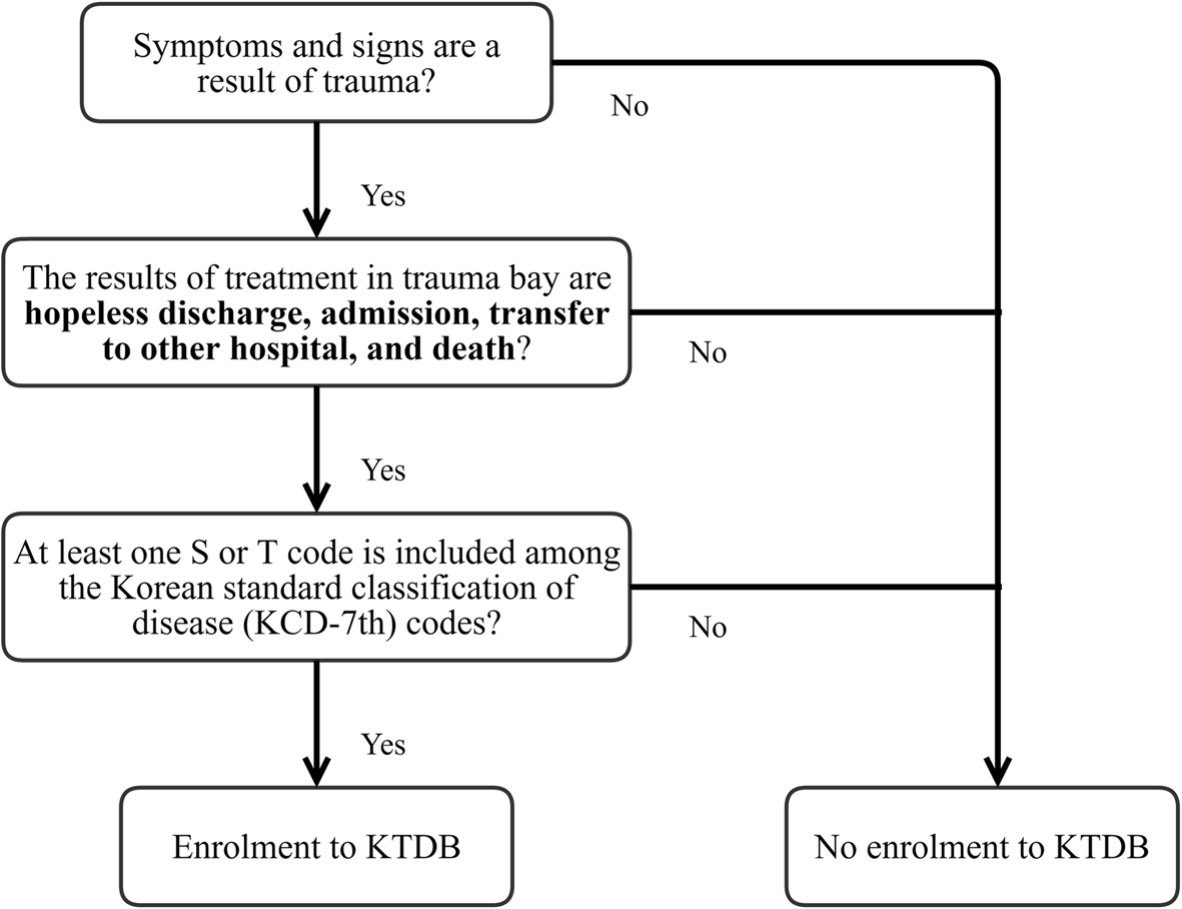
Criteria for enrolment in the Korean trauma data bank *KCD* Korean Standard Classification of Diseases, *KTDB* Korean trauma data bank

A list of all injured patients who visited the emergency department (ED) from 1 January 2018 to 31 August 2021 was collected to compare the number of visits before and after COVID-19. The Korean trauma database (KTDB) was used to collect medical data of trauma patients in the study. The KTDB, used to store prospectively collected data of trauma patients from each trauma centre hospital information system, is managed by the trauma project group (under the Ministry of Health and Welfare). Using the data from each trauma centre, the trauma project group constructs one dataset. Although it is not permitted that data collected from every trauma centres in Korea be used together for research purposes, data collected from one trauma centre can be used by the same trauma centre for research purposes. Therefore, medical datacollected from our hospital as part of the KTDB from 1 January 2018 to 31 August2021 were analysed retrospectively in this study. Patients whose treatment outcomesin the trauma bay included hopeless discharge, admission, transfer to other hospitals,and death, were included in the KTDB. Hopeless discharge was defined as the discharge of patients with almost no chance of recovery from the ED. This classificationwas based on a subjective criterion used in our hospital, and decided upon by the treating physician. In addition, patients with the diagnoses classified using the S or Tcodes, according to the Korean standard classification of disease codes, were enrolled in the KTDB. Diagnoses classified as S or T code means that it occurred becauseof an injury or certain other consequences of external causes [11]. Patients with minor conditions who were discharged after treatment from the ED were not enrolled. The KTDB enrolment are summarised in Fig. 1. This retrospective study was approved by the Institutional Review Board of Wonju Severance Christian Hospital (IRB no. CR321173). Informed consent was waived because the data were anonymously analysed.

Using the data bank, variables such as age, sex, initial vital signs, Glasgow coma scale (GCS) score, abbreviated injury scale (AIS) score, injury severity score (ISS), injury mechanism, intention of the injuries, origin of the ED visit, transportation for the ED visit, trauma team activation (TTA), admission department, department performing the operation, results of ED visit, time from injury to ED visit, duration of ED stay, time from injury to surgery, time from ED visit to operation, hospital length of stay (LOS), intensive care unit (ICU) LOS, duration of mechanical ventilation (MV), transfusion within 24 h, and mortality were analysed. The number of confirmed cases was also recorded [4].

### Trauma patients care policies in Wonju Severance Christian hospital during COVID-19 era

After the first case of COVID-19 in South Korea was confirmed on 20 January 2020, the Committee of Infection Control at our hospital established a policy to respond to COVID-19 in terms of the management of trauma patients. One of the important issues was how to deal with patients with mild fever (≥ 37.5 ℃) who visited the trauma centre. From 21 January 2020, stable trauma patients with high body temperature (BT) (≥ 37.5 °C) were immediately admitted to the isolation sector in the ED, and nasal swabbing was performed for the COVID-19 polymerase chain reaction (PCR) test. After completing the physical examination and nasal swab, the patients were admitted to the isolation ward immediately after visiting the trauma centre. Quarantine in isolation wards was continued until the results of PCR tests for COVID-19 were reported. The results of the PCR tests were reported twice a day, and it took at least 6 h to report. Unstable trauma patients with a high BT (≥ 37.5 °C) were immediately isolated to the isolation sector in the ED. Medical staff wearing level D protective suits performed the necessary procedures. If surgery or computed tomography (CT) was necessary for these patients, a negative-pressure isolation chamber was used. Operations were performed by surgeons and nurses wearing Level D protective suits and portable coolers. Since 26 February 2021, the policy changed slightly following commencement of the rapid PCR testing for COVID-19 (Xpert Xpress SARS-COV-2 test, Cepheid, USA). Therefore, trauma patients with a high BT (≥ 37.5 °C) were admitted to the isolation sector in the ED, and a rapid PCR test was performed. Until the results of the PCR test were obtained, they remained in the isolation sector.

### Trauma trends in Korea

According to the trauma registration system statistics from 2018 to 2020, the total number of patients who visited the regional trauma centers in Korea was 37,372, 37,635, and 34,318 from 2018 to 2020, respectively. The criteria for enrolment in the trauma registration system are similar to those of KTDB enrolment. The proportion of severely injured patients (ISS ≥ 16) was 22.2%, 23.6%, and 26%, respectively, from 2018 to 2020. Male dominated in the yearly sex proportion (73.5%, 65.2%, and 65.5%, respectively). The proportion of blunt trauma was higher than that of penetrating trauma from 2018 to 2020 (91.6% vs 6.4%, 92.3% vs 6.2%, and 91.8% vs 6.5%, respectively). Traffic collision was the most common mechanism and its proportion was 36.4%, 35.9, and 33.8%, respectively. These reports are available in the statistical yearbook of the National Emergency Medical Center [12].

### Study setting

The number of patients who visited the trauma centre, was counted within similar calendar period (20 January 2018 to 31 August 2019 vs 20 January 2020 to 31 August 2021). The correlation between the monthly number of patients and number of confirmed cases was analysed.

As the first confirmed case of COVID-19 affected our hospital policy and society, trauma patients were divided into two groups based on time periods (pre-COVID-19 and COVID-19) as of 21 January 2021. The characteristics and outcomes of patients who visited our trauma centre before and after COVID-19 were comparatively analysed. Propensity score matching (PSM) was performed to compare outcomes before and after COVID-19 in trauma patients with similar severity and vital signs.

### Statistical analysis

Continuous variables are presented as mean ± standard deviation and categorical variables as frequencies and percentages. Continuous variables were tested for normality using the Shapiro–Wilk test and compared using the Student’s t-test or the Mann–Whitney U test, as appropriate. Categorical variables were also compared using the chi-squared test and Fisher's exact test, as appropriate. Spearman’s rank correlation analysis was performed to investigate correlations between continuous variables. For PSM, logistic regression analysis was performed including age, sex, systolic blood pressure (SBP), diastolic blood pressure, GCS, AIS 1–6, and ISS, and the propensity score for the predicted probability of a patient visiting the trauma centre was estimated using a multivariable logistic regression model. Using the nearest neighbour matching method, the absolute values of the differences in the estimated propensity scores of all trauma patients in the pre-COVID-19 and COVID-19 eras were paired from the smallest to the largest. Statistical significance was set at *p* < 0.05. Statistical analysis was performed using the R statistical software (version 4.1.0; R Foundation for Statistical Computing, Vienna, Austria). GraphPad Prism software version 9.0.0 (GraphPad, San Diego, CA, USA) was used to generate some of figures.

## Results

### Patient enrolment

**Fig. 2:**
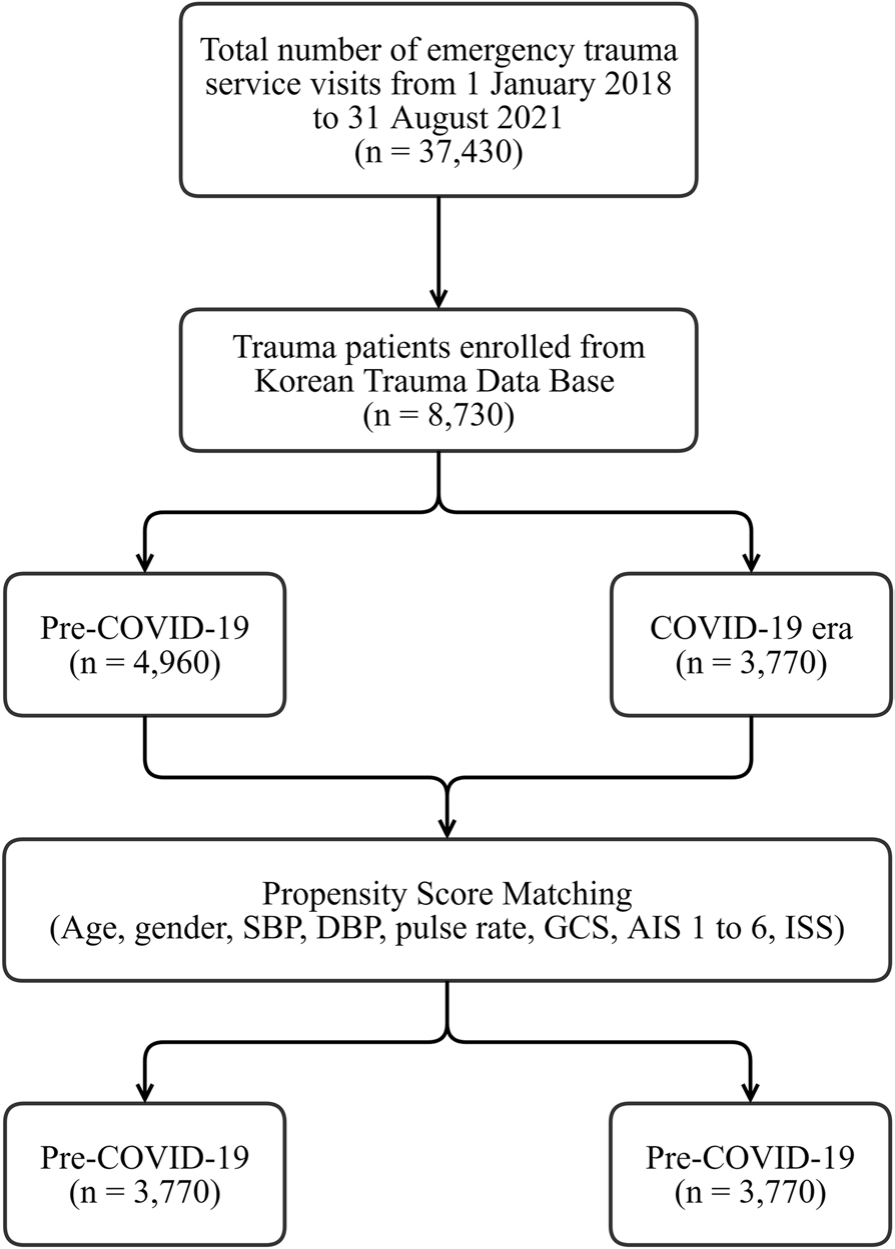
Patient flow chart *COVID* coronavirus disease, *SBP* systolic blood pressure, *DBP* diastolic blood pressure, *GCS* Glasgow Coma Scale, *AIS* abbreviated injury scale, *ISS* injury severity score

A total of 37,430 injured patients were managed by our emergency trauma service. In total, 8,730 patients were enrolled in the study. In the pre-COVID-19 and COVID-19 eras, there were 4,960 and 3,770 patients, respectively. After PSM, 3,770 patients each were enrolled in both time groups (Fig. 2).

### Trends in trauma centre visits

**Fig. 3:**
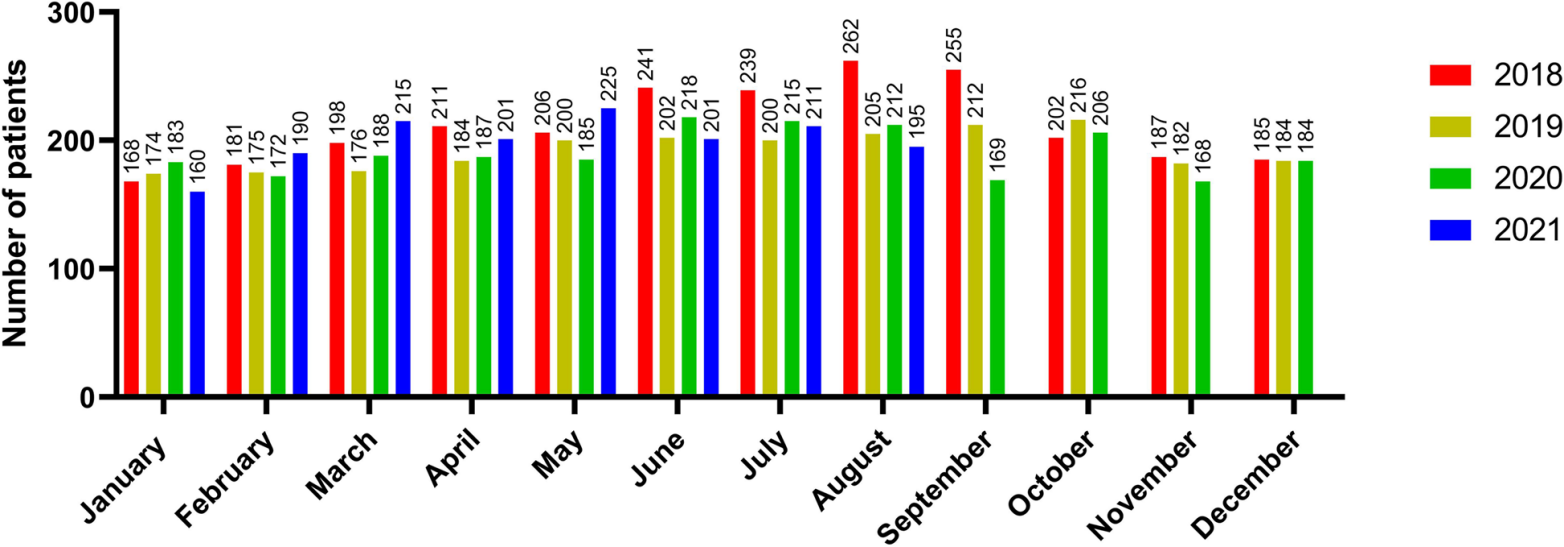
Differences in the number of patients monthly and annual visits

**Fig. 4:**
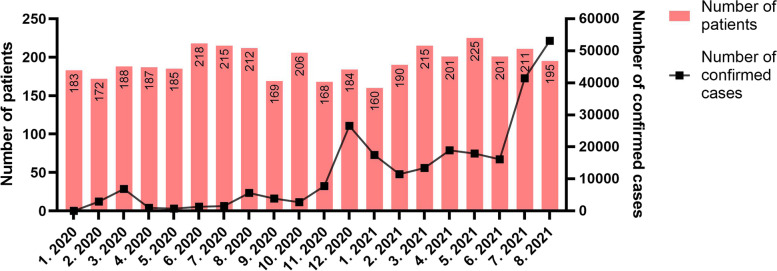
Monthly changes in the number of patient visits and number of confirmed cases

**Fig. 5:**
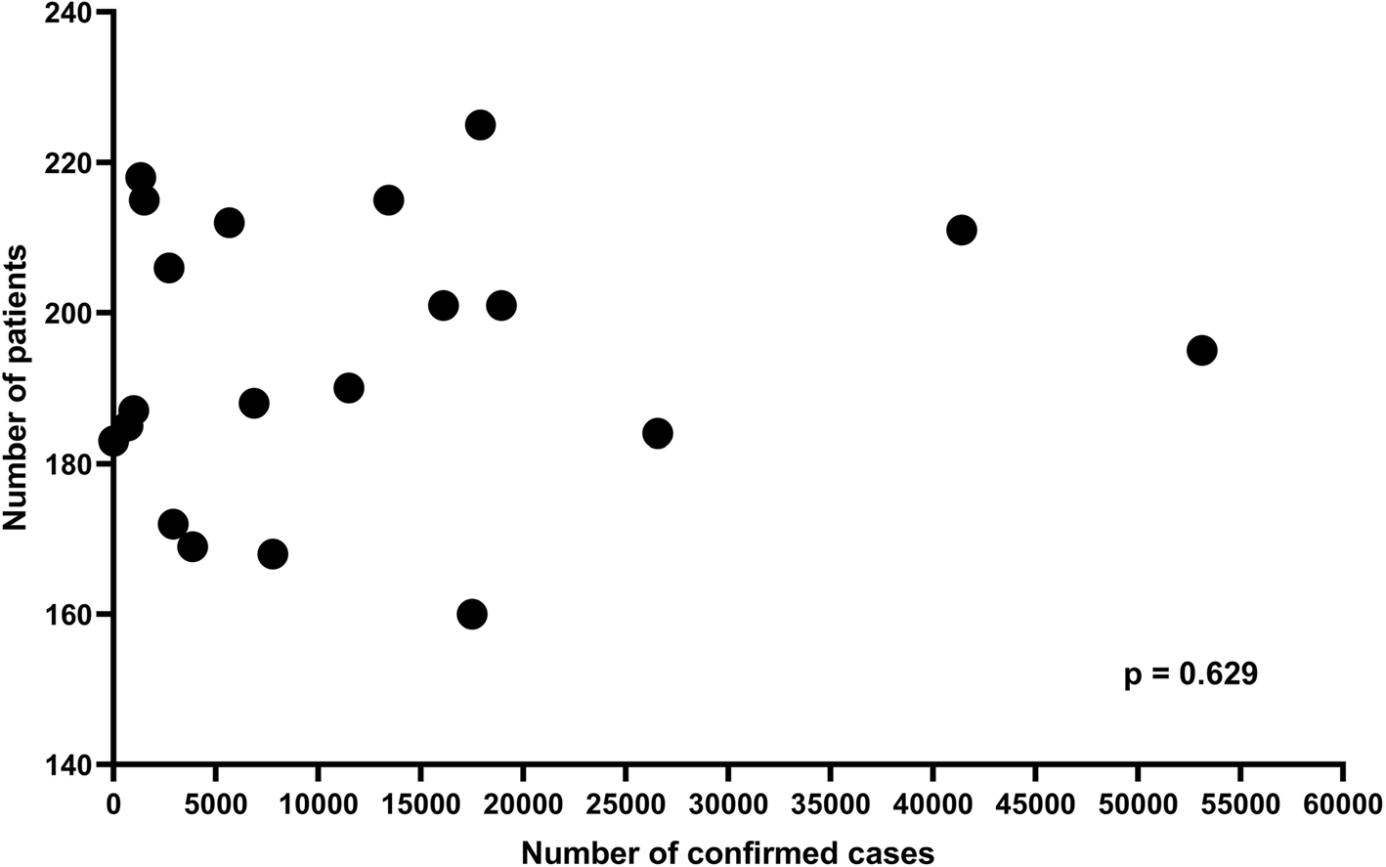
Scattered plot showing a no significant correlation between monthly number of patient visits to the trauma centre and monthly number of confirmed cases

The number of the injured patients decreased from 17,855 to 15,357; a decrease of 14.0% between the two periods (20 January 2018 to 31 August 2019 vs 20 January 2020 to 31 August 2021). The number of the injured patients included in the KTDB decreased from 3,944 to 3,770, a decrease of 4.4% between the same two periods. The annual differences in monthly visits are described in Fig. 3. The number of patients per month after the outbreak did not decline consistently. The patterns of the monthly number of patients visiting our trauma centre and the monthly number of confirmed cases after the COVID-19 outbreak are shown in Fig. 4. Scatter plots showed no significant correlation between the monthly number of patients visiting the trauma centre and the monthly number of confirmed cases (*p* = 0.629) (Fig. 5).

### Differences in the characteristics of trauma patients between the two time periods

Trauma patients who visited our trauma centre were significantly older in the COVID-19 era than pre-COVID-19 era (58.3 ± 20.8 vs 55.2 ± 21.5, *p* < 0.001). There was no significant difference in the proportion of male patients [2464 (65.4%) vs 3203 (64.5%), *p* = 0.462]. SBP (136.5 ± 37.7 vs 132.2 ± 39.3, *p* < 0.001) and pulse rate (82.7 ± 22.6 vs 81.4 ± 24.3, *p* = 0.011) were higher in COVID-19 era than pre-COVID-19. The proportion of patients with AIS1 ≥ 3 [781 (20.7%) vs 919 (18.5%), *p* = 0.011], AIS3 ≥ 3 [658 (17.5%) vs 751 (15.1%), *p* = 0.004], and AIS5 ≥ 3 [764 (20.3%) vs 873 (17.6%), *p* = 0.002] was higher in COVID-19 era than pre-COVID-19. The trauma mechanisms were significantly different between the two time periods (*p* < 0.001). The proportion of traffic accidents [1326 (35.2%) vs 1922 (38.8)] decreased in the COVID-19 era (compared to pre-COVID-19), while the proportion of falls increased [621 (16.5%) vs 752 (15.2%)]. Transportation for visits was significantly different between the two time periods (*p* < 0.001). Ambulance transportation increased in the COVID-19 era compared to pre-COVID-19 [2925 (77.6%) vs 3666 (73.9%)]. Age, SBP, pulse rate, AIS1, AIS3, AIS5, and ISS were adjusted for after PSM (Table 1).

**Table 1:** Characteristics of the patients who visited the trauma centre during pre-COVID-19 and COVID-19 era

	**Before PSM**			**After PSM**	
	**COVID-19 era (*n* = 3770)**	**Pre-COVID-19 (*n* = 4960)**	***p-*value**	**Pre-COVID-19 (*n* = 3770)**	***p-*value**
Age	58.3 ± 20.8	55.2 ± 21.5	< 0.001	58.3 ± 20.5	0.900
Sex (male)	2464 (65.4)	3203 (64.5)	0.462	2454 (65.1)	0.828
SBP (mmHg)	136.5 ± 37.7	132.2 ± 39.3	< 0.001	135.7 ± 36.8	0.351
DBP (mmHg)	77.2 ± 22.0	77.5 ± 23.7	0.629	77.5 ± 21.5	0.619
Pulse rate	82.7 ± 22.6	81.4 ± 24.3	0.011	82.6 ± 22.5	0.772
Body temperature (℃)	36.5 ± 2.9	36.4 ± 3.0	0.113	36.4 ± 2.5	0.491
GCS	13.7 ± 3.5	13.7 ± 3.7	0.591	13.6 ± 3.7	0.507
AIS (≥ 3)					
Head and neck	781 (20.7)	919 (18.5)	0.011	791 (21.0)	0.799
Face	19 (0.5)	26 (0.5)	1.000	22 (0.6)	0.754
Chest	658 (17.5)	751 (15.1)	0.004	671 (17.8)	0.717
Abdomen	271 (7.2)	320 (6.5)	0.189	274 (7.3)	0.929
Extremity and pelvic girdle	764 (20.3)	873 (17.6)	0.002	712 (18.9)	0.139
External	15 (0.4)	30 (0.6)	0.235	21 (0.6)	0.404
ISS	10.6 ± 9.3	9.6 ± 8.7	< 0.001	10.6 ± 9.1	0.941
Mechanism			< 0.001		< 0.001
Motor vehicle collision	1326 (35.2)	1922 (38.8)		1480 (39.3)	
Slip down	759 (20.1)	958 (19.3)		747 (19.8)	
Falls	621 (16.5)	752 (15.2)		595 (15.8)	
Other blunt trauma	496 (13.2)	577 (11.6)		418 (11.1)	
Penetrating trauma	143 (3.8)	245 (4.9)		170 (4.5)	
Others	425 (11.3)	506 (10.2)		360 (9.5)	
Intension of injury^a^			0.922		0.426
Non intentional	3399 (95.2)	4546 (95.4)		3467 (95.9)	
Suicide attempt	107 (3.0)	137 (2.9)		97 (2.7)	
Assault	63 (1.8)	81 (1.7)		57 (1.5)	
Origin of the visit			0.702		0.172
Transfer	1671 (44.3)	2220 (44.8)		1731 (45.9)	
Scene	2099 (55.7)	2740 (55.2)		2039 (54.1)	
Transportation for visit			< 0.001		0.119
Ambulance	2925 (77.6)	3666 (73.9)		2859 (75.8)	
Helicopter	134 (3.6)	193 (3.9)		161 (4.3)	
Private vehicle and walking	711 (18.9)	1101 (22.2)		750 (19.9)	

*PSM* propensity score matching, *COVID* coronavirus disease, *SBP* systolic blood pressure, *DBP* diastolic blood pressure, *GCS* Glasgow Coma Scale, *AIS* abbreviated injury scale, *ISS* injury severity score

^a^The intention of injury was unknown in 397 patients

### Department involved in patients’ management and TTA

The proportion of admission departments was significantly different between the two time periods (*p* < 0.001). The proportions of the admission to the department of Orthopaedic Surgery (OS) [1,108 (31.2%) vs 1,496 (33.5%)] and General Surgery (GS) [628 (17.7%) vs 976 (21.9%)] were lower in COVID-19 era compared to pre-COVID-19. There was no significant difference in the proportion of operating departments between the two time periods (*p* = 0.107). Trauma team was less frequently activated in COVID 19 era [1952 (51.8%) vs 2709 (54.6%), *p* = 0.009] (see Table 2). The TTA criteria used in our trauma centre are described in Additional file 1.

**Table 2:** Department involved patient’s management and trauma team activation

	**COVID-19 era (*n* = 3770)**	**Pre-COVID-19 (*n* = 4960)**	***p*-value**
Department of admission			< 0.001
OS	1108 (31.2)	1496 (33.5)	
NS	1007 (28.4)	1237 (27.7)	
GS	628 (17.7)	976 (21.9)	
CS	435 (12.3)	452 (10.1)	
EM	103 (2.9)	107 (2.4)	
Another department	269 (7.6)	198 (4.4)	
Total	3550	4466	
Department performed operation			0.107
GS	187 (39.3)	216 (37.2)	
NS	163 (34.2)	197 (33.9)	
OS	86 (18.1)	127 (21.9)	
CS	11 (2.3)	21 (3.6)	
Another department	29 (6.1)	20 (3.4)	
Total	476	581	
Trauma team activation	1952 (51.8)	2709 (54.6)	0.009

*OS* orthopaedic surgery, *NS* neurosurgery, *GS* general surgery, *CS* chest surgery, *EM* emergency medicine

### Comparison of factors associated with in-hospital intervention and patient outcomes before PSM

The results of the ED visits were significantly different between the two time periods (*p* < 0.001). Transfer to other hospitals decreased during COVID-19 era [164 (4.4%) vs 410 (8.3%)]. The time from injury to ED visits was significantly increased in the COVID-19 era compared to pre-COVID-19 (256.8 ± 338.3 vs 230.6 ± 317.7, *p* < 0.001). Hospital LOS (15.7 ± 22.9 vs 17.3 ± 28.0, *p* = 0.007) and duration of MV (4.2 ± 7.2 vs 6.6 ± 10.1, *p* < 0.001) were significantly decreased in the COVID-19 era compared to pre-COVID-19. The transfusion amount of red blood cell (0.6 ± 2.6 vs 0.6 ± 3.0, *p* = 0.930), fresh frozen plasma (0.5 ± 2.4 vs 0.5 ± 2.5, *p* = 0.701), and platelet concentration (0.1 ± 1.0 vs 0.1 ± 0.9, *p* = 0.219) was not different between the two groups. There was no significant difference in mortality between the two groups [209 (5.5%) vs (252 (5.1%), *p* = 0.363) (Table 3).

**Table 3:** Factors associated with in-hospital intervention and patient’s outcomes

	**Before PSM**			**After PSM**	
	**COVID-19 era (*n* = 3770)**	**Pre-COVID-19 (*n* = 4960)**	***p-*value**	**Pre-COVID-19 (*n *= 3770)**	***p-*value**
Result of ED visit			< 0.001		< 0.001
Admission to ward	2261 (60.0)	2914 (58.8)		2167 (57.5)	
Emergency operation	476 (12.6)	581 (11.7)		467 (12.4)	
Admission to ICU	813 (21.6)	971 (19.6)		803 (21.3)	
Transfer to other hospital	164 (4.4)	410 (8.3)		282 (7.5)	
Death in ER	56 (1.5)	84 (1.7)		51 (1.4)	
Time from injury to ED visit (minutes)	256.8 ± 338.3	230.6 ± 317.7	< 0.001	229.9 ± 313.9	< 0.001
ER stay (minutes)	280.5 ± 203.9	278.2 ± 223.9	0.614	278.4 ± 227.1	0.676
Time from injury to operation (minutes)	359.9 ± 254.9	366.5 ± 264.9	0.680	358.8 ± 258.5	0.947
Time from ED visit to operation (minutes)	203.1 ± 202.2	201.3 ± 197.0	0.883	193.9 ± 193.5	0.477
Hospital LOS (day)	15.7 ± 22.9	17.3 ± 28.0	0.007	18.6 ± 30.3	< 0.001
ICU LOS (days)	6.8 ± 11.4	7.3 ± 9.3	0.172	7.7 ± 9.7	0.031
Duration of mechanical ventilation (days)	4.2 ± 7.2	6.6 ± 10.1	< 0.001	6.9 ± 10.5	< 0.001
Transfusion within 24 h (units)					
Red blood cell	0.6 ± 2.6	0.6 ± 3.0	0.930	0.7 ± 3.1	0.235
Fresh frozen plasma	0.5 ± 2.4	0.5 ± 2.5	0.701	0.6 ± 2.6	0.488
Platelet concentration	0.1 ± 1.0	0.1 ± 0.9	0.219	0.1 ± 0.9	0.632
Mortality	209 (5.5)	252 (5.1)	0.363	196 (5.2)	0.540

*PSM* propensity score matching, *COVID* coronavirus disease, *ER* emergency room, *ICU* intensive care unit, *LOS* length of stay

### Comparison of factors associated with in-hospital intervention and patient’s outcomes after PSM

The results of the ED visits were significantly different between the two groups (*p* < 0.001). Transfer to other hospitals was increased during COVID-19 era [164 (4.4%) vs 282 (7.5%)]. The time from injury to ED visits significantly increased in the COVID-19 era compared to the pre-COVID-19 (256.8 ± 338.3 vs 229.9 ± 313.9, *p* < 0.001). Hospital LOS (15.7 ± 22.9 vs 18.6 ± 30.3, *p* < 0.001), ICU LOS (6.8 ± 11.4 vs 7.7 ± 9.7, *p* = 0.031), and duration of MV (4.2 ± 7.2 vs 6.9 ± 10.5, *p* < 0.001) significantly decreased in COVID-19 era than in pre-COVID-19 (Table 3).

## Discussion

The major finding of this study was the change in patient outcomes in the COVID-19 era. Hospital LOS and MV duration were significantly reduced during the COVID-19 era, which was different from our hypothesis. In addition to these two outcomes, ICU LOS was significantly lower in the COVID-19 era after adjustment for patient characteristics using PSM. The number of deaths did not differ between the two groups, although ISS increased during the COVID-19 era. Similar results have also been reported in other studies. DiFazio et al. reported a significant reduction in the hospital LOS [13]. Other studies also reported reduction of hospital LOS, ICU LOS, and ventilator days, although the findings were not statistically significant [7, 8]. Chiba et al. reported a significant reduction in ICU LOS and ventilator days [14]. This study demonstrated that the decrease in severe head trauma, chest trauma, and ISS during the pandemic could explain the significantly lower need for ICU LOS and ventilator use. However, in this study, the proportion of head and chest injuries (AIS ≥ 3) was higher in the COVID-19 era. Furthermore, ISS during the COVID-19 era was significantly higher in this study. A study conducted in other regional trauma centres in Korea demonstrated similar findings, showing significantly reduced hospital LOS with no change in ISS [15]. Korea’s health security system has two components: mandatory social health insurance and medical aid. The National Health Insurance (NHI) system provides healthcare coverage for all citizens. It is helpful to prevent catastrophic expenditure on health, leading to the traditional overuse of medical resources. In this context, trauma physicians may change their behaviour to reserve hospital resources during the COVID-19 pandemic. They may also change their mindset to plan for early discharge because healthcare facilities are considered one of the primary locations of virus transmission [16]; in hospitals, viral infection in severely injured patients may lead to more severe symptoms and worse prognosis. Furthermore, our hospital operated an isolation ward for patients with COVID-19 without additional manpower and prepared a part of the ICU with dedicated beds for patients. Therefore, the changes in hospital settings and physician attitudes in situations in which hospital resources were overused could have resulted in a decrease in hospital LOS, ICU LOS, and duration of MV, even between patients with similar severity and characteristics.

The results of the trauma centre visits showed an increase in general ward admissions and a decrease in transfers to other hospitals. Inter-hospital transfer has been limited as COVID-19 has spread in Korea due to the transfer guidelines of the Korean Medical Association and the Korean Society of Emergency Medicine. The transfer guidelines recommend that PCR for COVID-19 should be performed for patients with fever and respiratory symptoms if inter-hospital transfer is necessary [17]. As a regional trauma centre, trivial patients were transferred to primary or secondary medical institutions before the COVID-19 outbreak. The difficulty of transfer to other hospitals may lead to an increased general ward admission rate.

It was expected that the time from injury to surgery might be prolonged because patients were required to undergo an oropharyngeal swab test, and the time from injury to ED visits was prolonged. However, the time from injury to surgery was not prolonged during the COVID-19 era in this study. Xpert Xpress SARS-Cov-2 (Cepheid, USA), an automated diagnostic test for the qualitative detection of nucleic acids from SARS-CoV-2, was performed for patients who had fever before emergency surgery. The standard real-time PCR takes about 6–8 h, while the Xpert test takes about 30 min to 1 h on average, with excellent test performance [18]. This may help prevent delays in the treatment of injured patients who require emergency surgery.

In this study, we observed a decrease in the number of visits during COVID-19 era (14.0%) during similar calendar period between the pre-COVID-19 and COVID-19 eras. This decrease can be explained by several factors. First, the fear of being infected with COVID-19 in healthcare facilities may have had an impact on the behaviour of Koreans. In a survey conducted in Korea, 92.3% of the respondents answered that they avoided healthcare facilities as a means of social distancing [19]. Second, social distancing policies may have directly affected the incidence of injuries. In the same survey mentioned above, 96.7%, 87.4%, and 83.4% of participants responded that they avoided outdoor activities, public transportation, and crowded places, respectively [19]. This tendency to avoid travel and outdoor activities may result in a decrease in the number of injuries and visits. A study by Nunez et al. performed in Spain reported a significant reduction in the number of emergency trauma visits from March to April 2020 compared with the past [9]. A study from Netherlands also documented a 37% decrease in emergency trauma visits during same calendar period before and after COVID-19 outbreak [20]. A Saudi Arabia study reported 61% reduction in emergency trauma visit [21]. A South Africa study reported 47% reduction in the number of trauma cases [22]. The discrepancy in the reduction rate between our study and other studies may be due to several reasons. First, the strength of social restrictions differs between countries studies was conducted. Strict restrictions such as lockdowns have not been enforced in South Korea. Second, our results may reflect a relaxed social distancing policy during the study period and the resulting loose social atmosphere because our study period included the late period of the pandemic compared with other studies. The reduction in the number of visits based on the KTDB was smaller than that the reduction rate from overall patients who visited our trauma centre (4.4% vs 14.0%). This was because patients with more severe injuries were included in the KTDB. The significant prolongation of the time from injury to ED visit was in line with the reduction in trauma centre visits. Less injured patients with endurable symptoms may stay at home rather than visit the centre immediately because of fear of being infected with COVID-19, and if symptoms do not disappear, they may visit later. In addition, time may increase for transferred patients.

A higher ISS and a higher proportion of head/neck, chest, and extremity injuries were observed in the COVID-19 era. For the same reason mentioned above, less severely injured patients with a lower ISS may not visit the ED directly. In addition, this may be explained by a change in the mechanism. The proportion of injuries caused by falls increased during the COVID-19 era. Head injuries are commonly observed in falls regardless of fall height [23], and in particular, head/neck, chest, and extremity injuries are frequently related to falls in elderly patients [24]. The changes in AIS and ISS in this study may reflect changes in the mechanism of the injury. Studies have reported decreased ISS during the COVID-19 era [8, 13, 14]. The discrepancy in our results may be due to the difference in the degree of social restriction.

The proportion of motor vehicle collisions decreased in this study (38.8% vs 35.2), whereas the proportion of falls increased (15.2% vs 16.5%). This could be explained by the reduction in commuters and the recommendation to stay at home. Keays et al. reported a significant reduction in motor vehicle collisions during the COVID-19 pandemic [25]. A South Africa study reported that patients injured during traffic collisions decreased by 74% during the hard lockdown period and maintained a reduction of 32% during the immediate post-lockdown period [26]. Chiba et al. reported a significant increase in the number of falls [14].

In our trauma centre, fewer injured patients were admitted to the Department of OS and GS. Patients with polytrauma had lower injury severity, such as multiple contusions admitted to GS, if they wanted to be admitted to our centre. After the COVID-19 outbreak, trivial patients tended to be discharged in the ED and followed up in the outpatient clinic compared to the past. This tendency might have resulted in the decreased proportion of GS and OS admissions.

In this study, the frequency of TTA was reduced during the COVID-19 era. Leichtle et al. published results from a level 1 trauma centre in USA, where trauma activations dropped 43% compared with control groups during COVID-19 outbreak [27]. They suggested that this may be explained by the reduction in the occurrence of major trauma due to the official stay-at-home order from the state. However, the ISS increased in our study. We used TTA criteria based on the field triage, including trauma mechanism, which means the patients may be overtriaged by serious injury mechanisms such as automobile crashes. The decrease in motor vehicle collisions might affect the reduction in TTA.

It is well known that most trauma patients are usually working-age males [10]. They usually involve non-lockdown activities, such as road traffic crashes and work, school, and sports injuries [28]. Therefore, the mean age of the enrolled patients was older in the COVID-19 era than in the pre-COVID-19 period, as trauma related to non-lockdown activities may be reduced.

Increased transport to the centre by ambulance likely correlated with the observed increased ISS. Patients with more severe injuries are possibly more likely to be transported by ambulance.

This study had several limitations. First, it was conducted at a single trauma centre; however, our trauma centre is one of the core trauma centres covering the largest region of South Korea. Despite this, our results may not be generalisable to other trauma centres. Second, this was a retrospective observational study. Therefore, we may not have captured all the data needed to explain the changes in the trauma centre visits. Unrecognised factors other than COVID-19 may have affected the results of our study. However, it is almost impossible to conduct a prospective study to explore these changes during the pandemic. Given these circumstances, we performed a retrospective study using as much information as possible. Third, our study did not examine the number of patients with minor trauma who were discharged from the ER. Fourth, some important risk factors such as comorbidity and body mass index (BMI) could not be obtained from the database because comorbidity was not recorded in the early version of KTDB. BMI had not been recorded. Fifth, hopeless discharge, as a final disposition after ED treatment, was used, and the classification was based on a subjective criterion. Because it is not a widely used term, it may be difficult to compare this final disposition results with those of other similar studies. Finally, our hospital is not a designated referral hospital for burns. Therefore, selection bias was unavoidable. A nationwide or national multicentre study will be more meaningful to examine the impact of the COVID-19 outbreak on the change in trauma patterns, volume, and patient outcomes.

Despite these limitations, our study is meaningful because it showed accurately how patient outcomes due to hospital management have changed during the COVID-19 outbreak in similar patients whose characteristics and severity have been adjusted. In addition, this study may include the impact of long-lasting social distancing measures as the study period was longer.

## Conclusion

The number of ED trauma service visits has reduced. Furthermore, characteristics of the enrolled patients, such as age, AIS1, AIS3, AIS5, ISS, injury mechanism, and transportation for visits, were significantly different between the two groups. Admission to the general ward increases the time from injury to ED visits.

Interestingly, patient outcomes such as hospital LOS, ICU LOS, and duration of MV improved, while injury severity worsened during the COVID-19 era. After adjusting for patient characteristics and severity, similar findings were observed. The small change in the number of patient visits, and those with higher ISS highlight the importance of maintaining trauma service capacity and capability during the pandemic. It also could be another lesson for trauma centres in Korea after the endemic change of coronavirus that a shift in the mindset of trauma physicians will be needed to prevent overuse of traditional hospital resources and thereby reduce medical expenses.

## Supplementary Information

**Additional file 1. **Trauma team activationcriteria.

## Data Availability

The datasets generated and/or analysed during the current study are not publicly available due to privacy or ethical restrictions but are available from the corresponding author on reasonable request.
