# Supplementary material for: Outcomes improvement despite continuous visits of severely injured patients during the COVID-19 outbreak: experience at a regional trauma centre in South Korea
Source: BMC Emerg Med. 2022 Oct 6;22:167. doi: 10.1186/s12873-022-00726-1 (PMC9540133; doi:10.1186/s12873-022-00726-1)
Supplement: Supplementary file 1 — Additional file 1. Trauma team activationcriteria. [file 12873_2022_726_MOESM1_ESM.docx]

**Additional file 1**

**Additional file 1. Trauma team activation criteria**

1. Physiologic criteria for injured patients

A. Airway obstruction/respiratory failure

B. Intubated status before trauma centre arrival

C. Adults: respiratory rate <10 breaths/min or >30 breaths/min.

D. Adults: systolic blood pressure <90 mm Hg

E. Adults: heart rate >100 beats/min

F. Glasgow coma scale <13

2. Anatomical criteria

A. All penetrating injuries

I. Head and neck, chest, abdomen

II. Extremity: proximal to elbow or knee

B. Chest

I. Flail chest

C. Nervous system

I. Open or depressed skull fracture

II. Paralysis or suspected spinal cord injury

D. Extremity and pelvis

I. Pelvic bone fracture

II. Two or more proximal long-bone fractures

III. Crushed, degloved, mangled, or pulseless extremity

IV. Amputation proximal to the wrist or ankle

3. Mechanism of injury

A. Automobile crash: death in same passenger compartment

B. Automobile crash: ejection from automobile

C. Automobile crash >60 km/h

D. Automobile versus pedestrian injury >30 km/h

E. Time for evacuation of victims in automobiles >20 min (intrusion, including roof: >30 cm at any site).

F. Motorcycle, bicycle crash >30 km/h

G. Fall

I. Adults: >6 m

II. Children: >3 m

H. Injury from explosion

4. Attending trauma physician’s judgment

* The trauma team should be activated when a patient has more than one criterion.

* Trauma team activation is determined by the findings observed at the beginning of the patient’s visit.
